# Examination of Advanced Function and Its Correlates in a Cohort of Community-Dwelling Older Adults

**DOI:** 10.1093/geroni/igaa057.652

**Published:** 2020-12-16

**Authors:** Daniel Liebzeit, Wan-chin Kuo, Beverly Carlson, Kimberly Mueller, Lisa Bratzke

**Affiliations:** 1 William S. Middleton Memorial Veterans Hospital, Verona, Wisconsin, United States; 2 University of Wisconsin-Madison, Madison, Wisconsin, United States; 3 San Diego State University, San Diego, California, United States

## Abstract

Function in older adults includes multiple domains, from basic to “advanced,” but we remain limited in detection of advanced function (engagement in social, leisure, and productive activities). The objective is to describe advanced function and examine relationships with basic function and health outcomes in community-dwelling older adults aged 55-65 years. This is an analysis of existing data from a large, ongoing cohort study, The Wisconsin Registry for Alzheimer’s Prevention (WRAP R01 AG027161). We used a 1:1 prospective case–control design to examine whether older adults with lower advanced function (lower functioning group) at wave 3 showed lower IADLs and poorer health outcomes in wave 4, compared to those with higher advanced function (higher functioning group). The lower functioning group had a mean advanced function score of 74.4 (SD = 10.1), compared to 98.7 (SD = 11.1) in the higher functioning group. The mean IADL scores were similar in the two groups (p = 0.123). The lower functioning group had significantly lower self-rated health (mean = 3.52; SD = .79) than higher functioning group (3.70; 0.79) and a significantly lower proportion of individuals with unimpaired, stable cognition (77%) than the higher functioning group (85%). The lower functioning group had higher rates of comorbidities (4.25 vs. 3.96), mortality (4 vs. 1), and depressive symptoms (CES-D: 7.17 vs. 6.09), although results were not significant at α=0.05. This study provides a foundation for examining advanced function, which may be an early indicator of poor health outcomes in older adults.

